# Supplementary material for: SKI-1/S1P Facilitates SARS-CoV-2 Spike Induced Cell-to-Cell Fusion via Activation of SREBP-2 and Metalloproteases, Whereas PCSK9 Enhances the Degradation of ACE2
Source: Viruses. 2023 Jan 27;15(2):360. doi: 10.3390/v15020360 (PMC9959508; doi:10.3390/v15020360)
Supplement: Supplementary file 1 [file viruses-15-00360-s001.zip › viruses-2120986-supplementary.pdf]

Supplementary FIGURE LEGENDS

Figure S1. (**A**) Schematic representation of human pro-BDNF and it cleavage sites by SKI-1 and Furin (arrows). SKI-1 cleaves the proBDNF at **R**GL**T**$_{57}$↓ generating a 28 kDa N-terminal fragment, whoever, furin generates mature BDNF directly from pro-BDNF or from the 28 kDa intermediate by cleaving at **R**V**RR**$_{128}$↓. (**B**) In HeLa cells cDNA encoding proBDNF was co-expressed with an empty vector (EV) or one encoding furin or SKI-1. Proteins from 48h conditioned media were resolved on 8% SDS-PAGE and analyzed using an anti-BDNF antibody (ANT-010, Abmone Labs). The molecular masses and the generated fragments are indicated.

Figure S2. In the presence of ACE2, SKI-1 enhances the cleavage of proS at S2". (**A**) Hela cells were transfected with a cDNA empty vector (EV) alone or with one encoding WT-S glycoprotein or its furin-resistant mutants (µS1/S2, µAS1/S2) in the presence or absence of SKI-1. (**B**) Hela cells transiently expressing ACE2 and WTS glycoprotein or its Ala-mutants (R765A, R847A). At 24h post transfection the cells were treated or not with a furin-like inhibitor BOS-981 at 3 µM. At 48h post transfection the cell extracts were analyzed by Western blot using mAb-V5.

Figure S3. ACE2 and less so LDLR act as SARS-CoV-2 receptors. Donor HeLa cells expressing empty vector (EV) and/or the S-glycoprotein were co-cultured for 18h with acceptor TZM-bl cells expressing (**A**) LDLR or a combination of ACE2 and LDLR. (**B**) During the co-culture cells were incubated with conditioned media from HEK293 cells expressing an empty vector or WT PCSK9. The extent of fusion is represented as relative luminescence units (RLU). Representative data of at least three independent experiments are shown. P values (****, $p < 0.0001$) were evaluated by a Student's t-test.

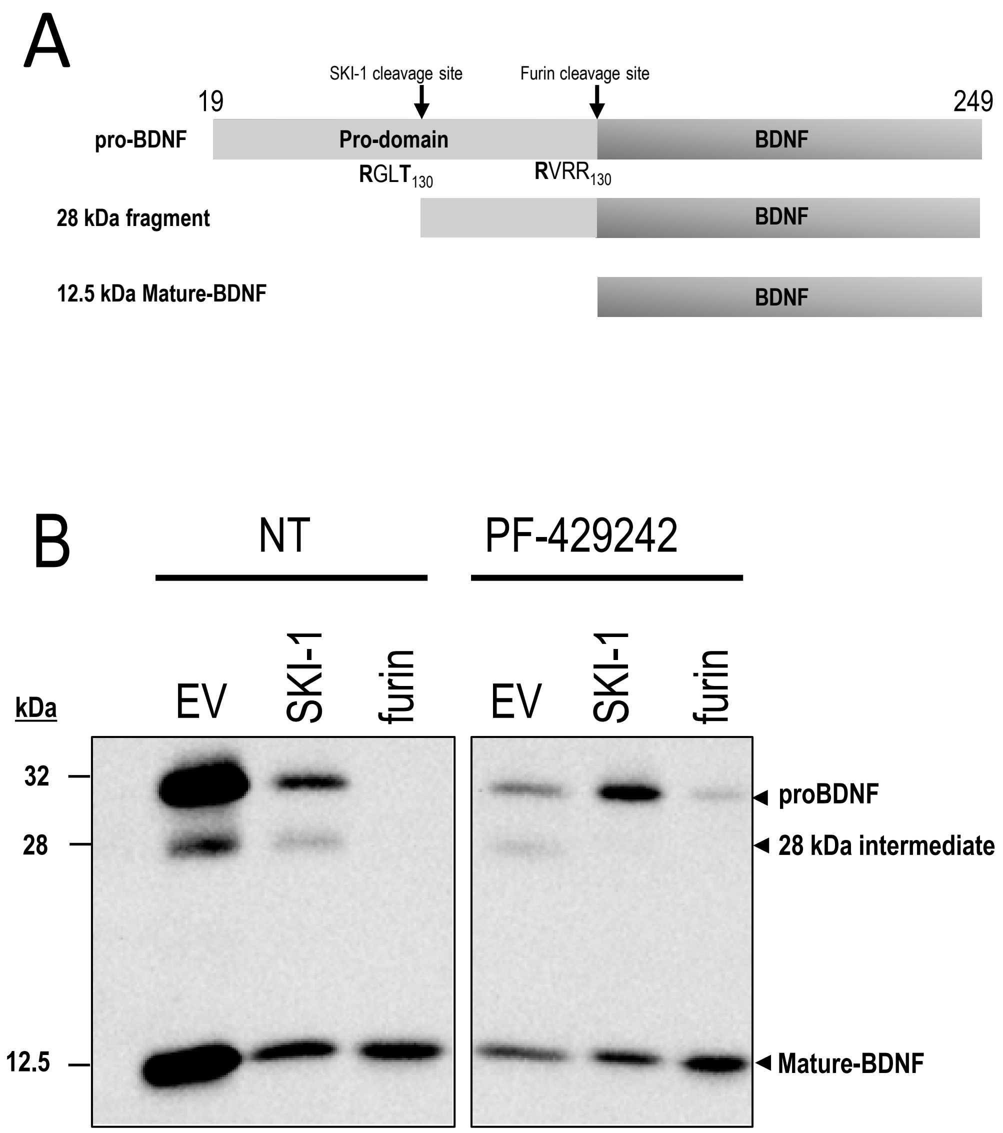
A
SKI-1 cleavage site
Furin cleavage site
19
249
pro-BDNF
Pro-domain
BDNF
RGLT130
RVRR130
28 kDa fragment
BDNF
12.5 kDa Mature-BDNF
BDNF
B
NT
PF-429242
kDa
EV
SKI-1
furin
EV
SKI-1
furin
32
28
12.5
proBDNF
28 kDa intermediate
Mature-BDNF

Figure S1

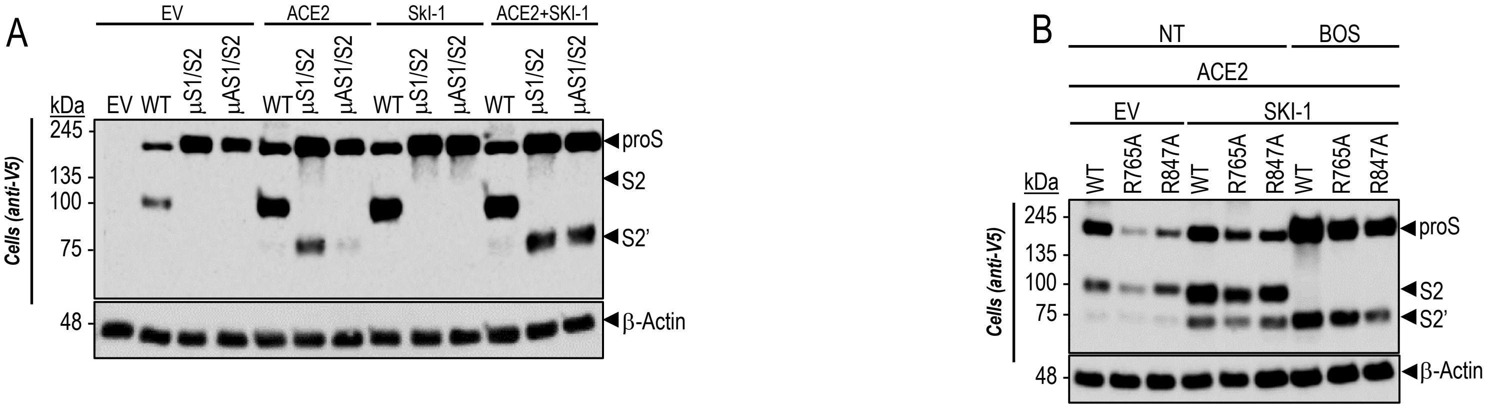
A
EV
ACE2
SkI-1
ACE2+SKI-1
kDa
EV
WT
µS1/S2
µAS1/S2
WT
µS1/S2
µAS1/S2
WT
µS1/S2
µAS1/S2
WT
µS1/S2
µAS1/S2
245
135
100
75
48
Cells (anti-V5)
proS
S2
S2'
β-Actin
B
NT
BOS
ACE2
EV
SKI-1
kDa
WT
R765A
R847A
WT
R765A
R847A
WT
R765A
R847A
245
135
100
75
48
Cells (anti-V5)
proS
S2
S2'
β-Actin

Figure S2

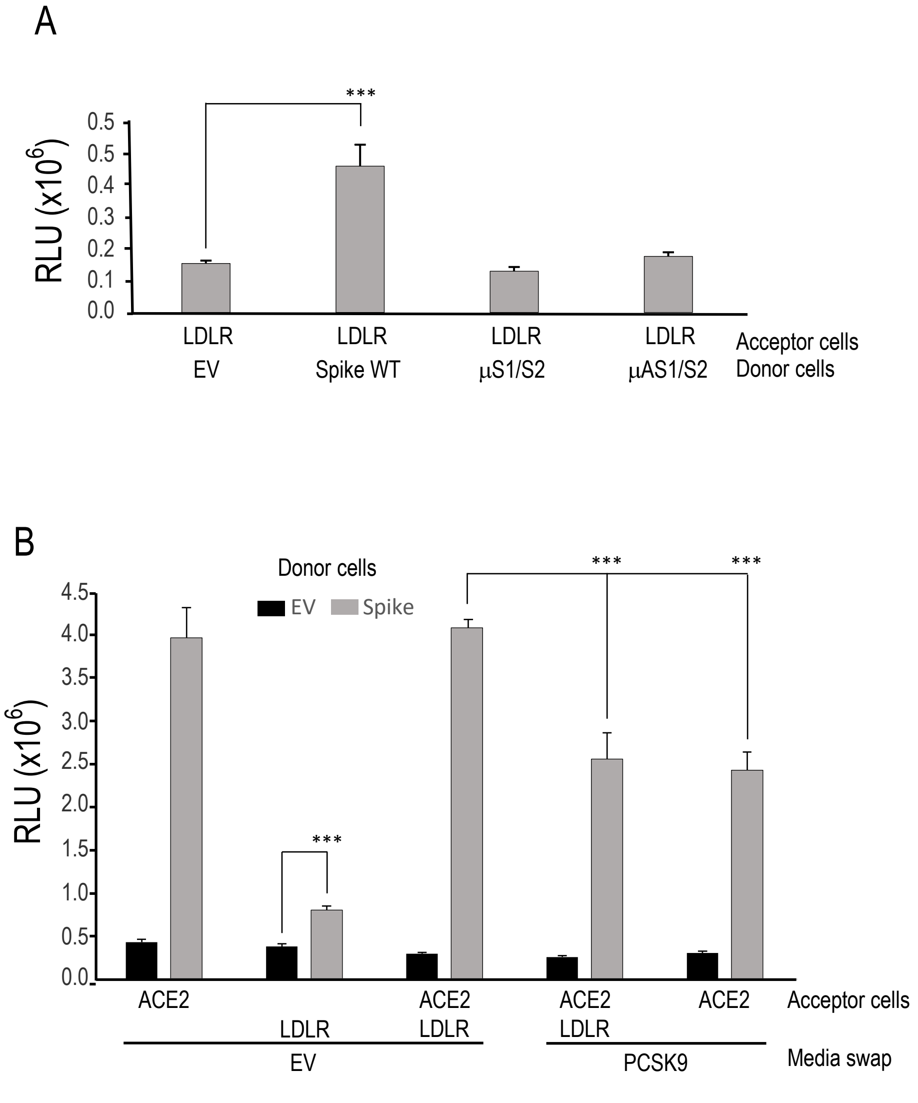
A
RLU (x10^6)
0.5
0.5
0.4
0.3
0.2
0.1
0.0
***
LDLR
EV
LDLR
Spike WT
LDLR
μS1/S2
LDLR
μAS1/S2
Acceptor cells
Donor cells
B
Donor cells
EV
Spike
RLU (x10^6)
4.5
4.0
3.5
3.0
2.5
2.0
1.5
1.0
0.5
0.0
***
***
***
ACE2
LDLR
ACE2
LDLR
ACE2
LDLR
ACE2
Acceptor cells
EV
PCSK9
Media swap

Figure S3
